# Supplementary material for: Repeated passive visual experience modulates spontaneous and non-familiar stimuli-evoked neural activity
Source: Sci Rep. 2023 Nov 27;13:20907. doi: 10.1038/s41598-023-47957-1 (PMC10684504; doi:10.1038/s41598-023-47957-1)
Supplement: Supplementary file 1 — Supplementary Figure S1. [file 41598_2023_47957_MOESM1_ESM.pdf]

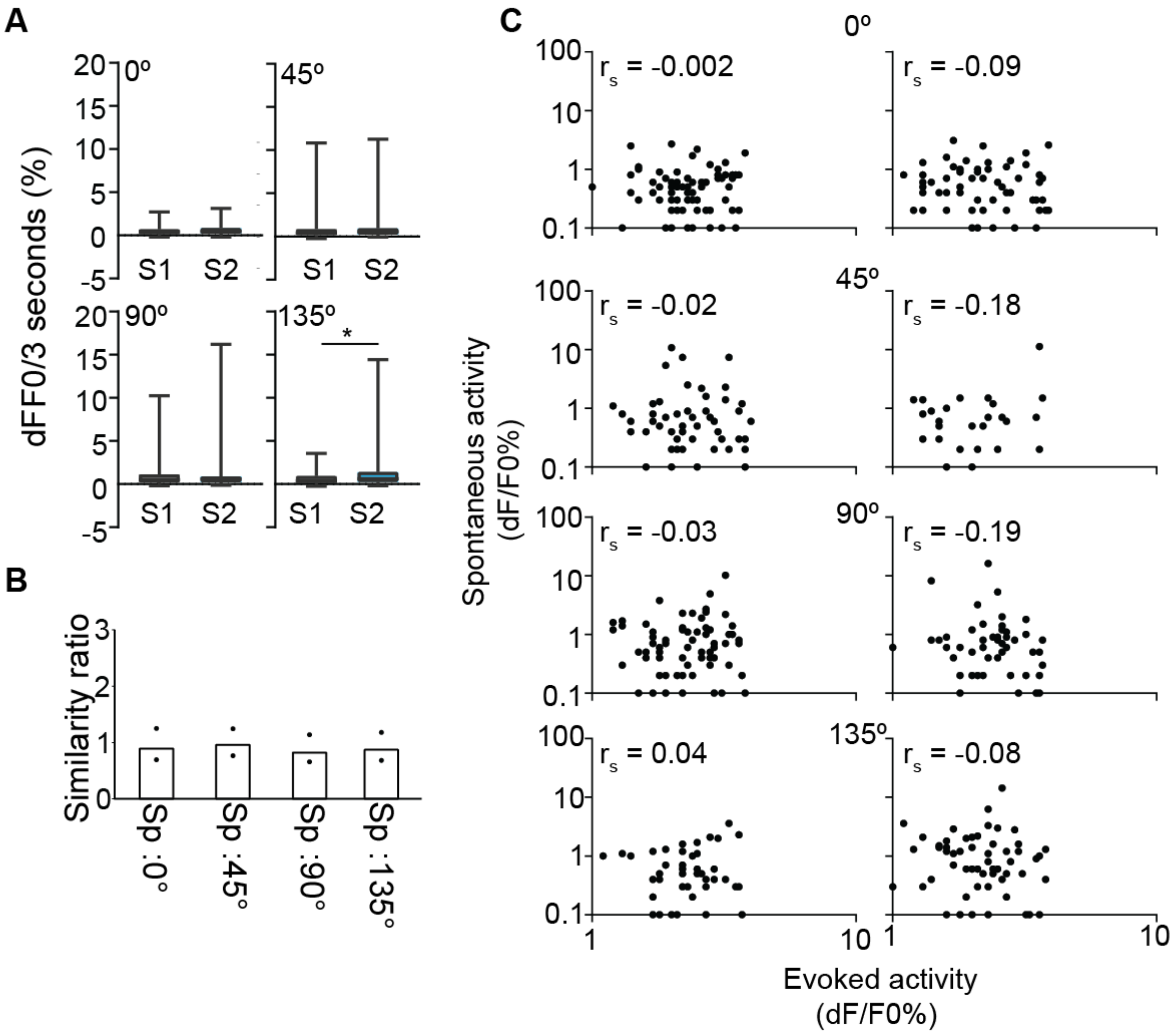


**Figure S1**. **Spontaneous and familiar stimulus-driven activity patterns in low responsive neurons. A**. dF/F0 averaged over three seconds during spontaneous activity in low-responsive neurons tuned to the indicated orientation before (S1) and after the altered experience (S2). Data are presented as box (25th to 75th percentile) and whisker (minimum and maximum values) plots, with the median value indicated as a horizontal line. n = 88, 64, 81, 53 (S1) and 69, 32, 54, 65 (S2) neurons tuned to 0˚, 45˚ (top), 90˚, 135˚ (bottom) bins, respectively from 9 mice. *$p$ < 0.05, linear mixed-effects model. **B.** Fold-change in the similarity of responses to spontaneous activity and evoked activity elicited by indicated grating stimulus or gray screen (GS) in neurons that are low responsive to any grating stimulus. $n$ = 286 (S1) and 220 (S2) neurons from 9 mice.

The dotted line indicates identical similarity after 45˚ experience. Black circles - the lower and upper bound of 95% confidence intervals (CI) obtained by bootstrapping with replacement. **C.** Spearman correlation ($r_s$) between average evoked dF/F0 and average spontaneous dF/F0 for low responsive neurons tuned to 0˚,  45˚, 90˚ or 135˚ (n = 88 (0˚) 64 (45˚), 81 (90˚), and 53 (135˚) neurons on S1 and 69 (0˚), 32 (45˚), 54 (90˚), and 65 (135˚) neurons on S2.
